# New Jersey State Veterinary Society

**Published:** 1893-03

**Authors:** A. T. Sellers

**Affiliations:** Secretary


*New Jersey State Veterinary Society.*—The New Jersey State Veterinary Society held its semi-annual convention at Saenger Hall, in the city of Newark, N. J., on Thursday, February 2d, with the President, Dr. J. D. Hopkins, in the chair. There was a good attendance of members from various parts of the State.

The minutes of the previous meeting were read and approved.

The President gave an instructive address on the duties of veterinarians and his usefulness as a sanitarian ; also words of good cheer as to the future of the profession, claiming that in the near future our services will be more fully appreciated than at present. Regretting very much that there seemed to be a lack of brotherly feeling among veterinarians, he hoped soon to see us working as one person, and striving for the elevation of our profession.

After hearing the reports of the Secretary, Treasurer and Board of Censors, the election of new members, the application for membership, the Society passed to the reading and discussion of papers. Dr. R. L. Tucker, of Plainfield, read a paper on "Bronchitis in the Dog." Dr. J. D. Hopkins one on "Differential Diagnosis between Osteo Porosis and Rheumatism." Both papers were freely discussed; quite a difference of opinion was manifested, especially as to Dr. Hopkins' statement that rheumatism did not exist in the adult horse. After parting with the discussion of the papers, the subject of "Foot Wounds" was taken up, and the discussion was only at its beginning when, owing to the lateness of the hour, the Society was compelled to adjourn.

A hearty vote of thanks was tendered to the essayists.

Adjourned to meet in annual session in the city of Newark.

A. T. SELLERS, D.V.S., *Secretary.*

